# Prognostic Value of a Stemness Index-Associated Signature in Primary Lower-Grade Glioma

**DOI:** 10.3389/fgene.2020.00441

**Published:** 2020-05-05

**Authors:** Mingwei Zhang, Xuezhen Wang, Xiaoping Chen, Feibao Guo, Jinsheng Hong

**Affiliations:** ^1^Department of Radiation Oncology, The First Affiliated Hospital of Fujian Medical University, Fuzhou, China; ^2^Institute of Immunotherapy, Fujian Medical University, Fuzhou, China; ^3^Key Laboratory of Radiation Biology (Fujian Medical University), Fujian Province University, Fuzhou, China; ^4^Fujian Key Laboratory of Individualized Active Immunotherapy, Fuzhou, China; ^5^Fujian Medical University Union Hospital, Fuzhou, China; ^6^Department of Statistics, College of Mathematics and Informatics & FJKLMAA, Fujian Normal University, Fuzhou, China

**Keywords:** lower grade glioma, The Cancer Genome Atlas, Chinese Glioma Genome Atlas, stemness indices-related signature, prognosis

## Abstract

**Objective:**

As a prevalent and infiltrative cancer type of the central nervous system, the prognosis of lower-grade glioma (LGG) in adults is highly heterogeneous. Recent evidence has demonstrated the prognostic value of the mRNA expression-based stemness index (mRNAsi) in LGG. Our aim was to develop a stemness index-based signature (SI-signature) for risk stratification and survival prediction.

**Methods:**

Differentially expressed genes (DEGs) between LGG in the Cancer Genome Atlas (TCGA) and normal brain tissue samples from the Genotype-Tissue Expression (GTEx) project were screened out, and the weighted gene correlation network analysis (WGCNA) was employed to identify the mRNAsi-related gene sets. Meanwhile, the Gene Ontology and Kyoto Encyclopedia of Genes and Genomes enrichment analyses were performed for the functional annotation of the key genes. ESTIMATE was used to calculate tumor purity for acquiring the correct mRNAsi. Differences in overall survival (OS) between the high and low mRNAsi (corrected mRNAsi) groups were compared using the Kaplan Meier analysis. By combining the Lasso regression with univariate and multivariate Cox regression, the SI-signature was constructed and validated using the Chinese Glioma Genome Atlas (CGGA).

**Results:**

There was a significant difference in OS between the high and low mRNAsi groups, which was also observed in the two corrected mRNAsi groups. Based on threshold limits, 86 DEGs were most significantly associated with mRNAsi via WGCNA. Seven genes (*ADAP2*, *ALOX5AP*, *APOBEC3C*, *FCGRT*, *GNG5*, *LRRC25*, and *SP100*) were selected to establish a risk signature for primary LGG. The ROC curves showed a fair performance in survival prediction in both the TCGA and the CGGA validation cohorts. Univariate and multivariate Cox regression revealed that the risk group was an independent prognostic factor in primary LGG. The nomogram was developed based on clinical parameters integrated with the risk signature, and its accuracy for predicting 3- and 5-years survival was assessed by the concordance index, the area under the curve of the time-dependent receiver operating characteristics curve, and calibration curves.

**Conclusion:**

The SI-signature with seven genes could serve as an independent predictor, and suggests the importance of stemness features in risk stratification and survival prediction in primary LGG.

## Introduction

Lower grade glioma is one of the prevalent and infiltrative types of primary malignant intracranial tumors in adults, the main components of which are diffuse low-grade and intermediate-grade gliomas (Ceccarelli et al., 2016; Ostrom et al., 2018). Despite comprehensive regimens that involve maximum surgical resection and subsequent radiotherapy and chemotherapy, the prognosis of LGG has not improved in the past four decades (Claus et al., 2015). Due to the great intrinsically biological and clinical heterogeneity, the overall survival (OS) of LGG estimates a range from 1 to 15 years, and the response to standard treatment varies from person to person (Cancer Genome Atlas Research et al., 2015). Although the histopathological classification of LGG has traditionally used to predict clinical outcomes, there remains a high intraobserver and interobserver variability, and is often hard to accurately predict outcomes even within the same grade (Coons et al., 1997; van den Bent, 2010). Therefore, it is imperative to search for novel molecular biomarkers for LGG genetic classification. Recently, the 2016 WHO brain tumor classification established the molecular markers for subclassification, including the chromosomal 1p and 19q (chr1p/19q) co-deletion, the isocitrate dehydrogenase (IDH) mutation, and the histone 3 mutational status. However, it seems that these widely utilized biomarkers have provided useful but insufficient prediction for risk stratification of patients with LGG, especially in genetically heterogeneous populations. Thus, novel prognostic parameters are urgently needed to develop and improve the stratification of LGG with the use of multiple advanced molecular platforms.

The complexity and heterogeneity of glioma cells is not only related to its genetic polymorphisms, but also to the characteristics of the microenvironment, such as stemness features and oncogenic and tumor suppressive pathways (Venteicher et al., 2017; Dirkse et al., 2019). Recent advancements have revealed that the populations of glioma stem-like cells are associated with the radio- and chemo-resistance, and with prognosis and tumor recurrence (Yi et al., 2016; Roos et al., 2017). To our knowledge, stemness features have been extracted by the novel stemness indices, including DNA methylation-based stemness index (mDNAsi), mRNA expression-based stemness index (mRNAsi) (Malta et al., 2018). Besides, Pan et al. (2019) developed a 13-gene prognostic signature based on mRNAsi, which suggested the stemness of cancer stem cells (CSCs) and the unfavorable prognosis. However, no study has previously attempted to identify the prognostic and predictive value of stem cell-related genes in LGG.

The scores of mRNAsi in LGG were computed using a one-class logistic regression machine learning algorithm (OCLR), and Tathiane et al. found a strong relationship between mRNAsi and prognosis of glioma, which provided new insights into stratification tumors with distinct clinical outcomes (Malta et al., 2018). However, that study mainly focused on comprehensive pan-cancer analysis. Despite the significant association observed between mRNAsi and OS, however, it was investigated based only on the level of bulky tumor. It is reasonable to take the tumor purity into account in order to further investigate the prognostic value of the stemness index in tumor parenchyma. In addition, a series of genes related to mRNAsi have not been analyzed in detail, and their biological function is also unknown. Meanwhile, the univariable and multivariable survival analyses of predominant clinicopathological factors (age, gender, IDH status, radiation, and chemotherapy status, etc.) and genes related to mRNAsi have not been explored in different cohorts. In order to identify the genes related to mRNAsi, the weighted gene correlation network analysis (WGCNA) was employed. This method takes the interrelation of genes into account for structure generation, instead of regarding genes as single entities. WGCNA has been applied to identify trait-related preserved modules for discovering the key genes (Zhang and Horvath, 2005; Langfelder and Horvath, 2008; Liang et al., 2019).

In addition, the ESTIMATE (Estimation of Stromal and Immune cells in MAlignant Tumor tissues using Expression data) algorithm is one of the most common methods to calculate the tumor purity, and is based on scores related to the level of immune cells infiltration and stromal cells in tumor tissues (Yoshihara et al., 2013). In the current study, the primary purpose was to identify the prognostic value of high- and low-score groups based on the mRNAsi or mRNAsi/purity in a Kaplan-Meier survival analysis. Next, differentially expressed genes (DEGs) were screened from The Cancer Genome Atlas (TCGA) database and the Genotype-Tissue Expression (GTEx) database. Subsequently, the WGCNA was applied for identifying the hub gene clusters and for selecting the stemness indices associated key genes in LGG. Meanwhile, the Gene Ontology and Kyoto Encyclopedia of Genes and Genomes (KEGG) enrichment analysis was employed for function annotation. Finally, the stemness-index associated gene signature was established and validated in the TCGA database and the Chinese Glioma Genome Atlas (CGGA) database, which were used for internal and external validation, respectively.

## Materials and Methods

### Data Source

The high-throughput RNA-seq data of 529 patients with LGG from the TCGA database and 1,152 normal brain tissue samples from the GTEx project were downloaded from the University of California Santa Cruz (UCSC) Xena website^1^. The gene expression profiles were quantified by fragments per kilobase of transcript per million mapped reads (FPKM) normalized estimation and log2-based transformation. Next, DEGs were selected by the “limma” package of R software under the threshold of absolute value of the log2-transformed fold change (FC) > 1 and the adjusted *P*-value (adj.*P*) < 0.05. Besides, the ComBat method was performed to remove the batch effects using the R package “sva.”

### Acquisition of Stemness Index Based on RNA-Seq

Malta et al. (2018) provided a novel analysis for an oncogenic dedifferentiation evaluation that considered the mRNAsi. The mRNAsi scores of the LGG samples were calculated when a one-class logistic regression machine learning algorithm (OCLR) was applied to LGG datasets from TCGA. The gene expression-based stemness index was represented using β values ranging from zero (no gene expression) to one (complete gene expression). The mRNAsi was obtained from the multiplatform analysis based on this previous research.

### Weighted Gene Correlation Network Analysis for Building Stemness-Index Associated Preserved Modules

The WGCNA was developed to discover the correlations among genes by constructing significant modules. The WGCNA analysis was performed by the “WGCNA package” for R (version 1.61)^2^ (Langfelder and Horvath, 2008).

Initially, the LGG transcriptome in the TCGA database was taken as a data source. The correlation of the expression levels of 5490 DEGs was analyzed with high precision and accuracy, which was a prerequisite for a WGCNA network development. Next, a parameter β was set based on the correlations of each DEG, which contributed to achieve a scale-free co-expression network. Next, the “blockwiseModules” function was carried out for constructing the network and detecting modules. Furthermore, the relationship between the modules and mRNAsi score was investigated, and the preserved module was determined by the top ranked modules with the strongest connections.

Finally, the key genes from the preserved module were explored. The Inclusive criterion for screening key genes was as follows: correlation (cor.) Gene GS > 0.5 and cor. Gene MM > 0.8 (Pan et al., 2019). Gene significance (GS) was calculated to measure the correlation between genes and sample traits (the values of mRNAsi), and Module Membership (MM) was used to assess the correlation between gene expression profiles and module eigengene. The associations among eigengenes, MM, and sample traits were assessed by Pearson’s correlation.

### Evaluation and Bioinformatics Analysis of Key Genes

The different expression levels of each key gene were visualized in a heatmap, which was retrieved from the normal tissue and tumor tissue. In addition, the interactions among key genes was visualized in a heatmap based on correlations. Moreover, the identification of the functional annotation was another vital step in the exploration of the potential mechanism of key genes. Thus, gene ontology (GO) enrichment analysis (Gene Ontology Constorium, 2015 and Kyoto Encyclopedia of Genes and Genomes (KEGG) (Kanehisa and Goto, 2000; Wanggou et al., 2016) signaling pathways were performed on a list of key genes. The visualization of results was implemented with the R “ggplot2” package. A *P* value < 0.05, and a false discovery rate (FDR) < 0.05 were considered to determine statistical significance.

### Inclusive and Exclusive Criteria of Enrolled Patients for the Construction of the Risk Signature

Inclusion criteria included: (1) patients who suffered from primary LGG (except for recurrent LGG), (2) complete clinicopathological feature, (3) diagnosed with WHO grade II or III glioma, (4) the RNA-sequencing data of samples was available, (5) the OS was set as the primary endpoint, and (6) patients with a minimum follow-up of 90 days.

The exclusive criteria were as follows: (1) patients with a pathological diagnosis of recurrence LGG, (2) patients who suffered from brain tumors other than LGG, and (3) absent survival status and clinicopathological parameters.

### Survival Analysis of mRNAsi

ESTIMATE, an algorithm based on a web tool^3^ provided information for the purity of the tumor tissue calculation (Yoshihara et al., 2013). The data of mRNA expression-based stemness index was calculated for each sample, and the Kaplan Meier analysis for samples with the high and low mRNAsi set was carried out. In view of the effects of tumor purity on the corresponding mRNAsi, the corrected mRNAsi (mRNAsi/tumor purity) was included. From another perspective, the survival rate between the high and low mRNAsi groups was re-compared using a Kaplan Meier analysis based on the corrected mRNAsi scores.

### Construction of a Prognostic Signature

A univariate Cox regression analysis was performed by the “survival” package in R to identify genes that are highly associated with and crucial for survival. The prognostic key genes were then further optimized by the least absolute shrinkage and selection operator (LASSO) regression model, using the R package “glmnet.” After completing the variable selection and the shrinkage of prognostic key genes, a stepwise multivariate Cox regression analysis was performed to generate the risk score model. The following formula was built based on the coefficients and expression levels for each gene.

$$\mathrm{Model}:R\,i\,s\,k\,s\,c\,o\,r\,e=\sum_{i=1}^{k}\beta \,i\,S\,i$$

Where *k* indicates the number of signature genes, β is equal to the coefficient index, and Si represents the expression level of key genes.

Afterward, using the “survminer” package in R (Li et al., 2019), the optimum cutoff value was obtained, and the primary LGG patients in the TCGA database were clustered into high-risk and low-risk groups. The gap of survival rates between the two groups was tested by the Kaplan–Meier analysis. The time dependent ROC was plotted in order to determine whether the risk score can accurately predict the survival status. Finally, the expression distributions of signature genes were shown in a heatmap using the “ComplexHeatmap” R package. The risk plot showed that the LGG patients in the TCGA database sorted by the rank of corresponding risk score.

### Prognostic Value of the Seven-Gene-Based Signature

The patients suffering from primary LGG in the TCGA dataset were randomly categorized into the training group (accounting for 70%) and internal validation group (accounting for 30%) by using the “caret” package^4^. The risk scores and the corresponding clinical variants, including age, gender, grade, radiotherapy, chemotherapy, and IDH status were subjected to univariate and multivariate Cox model. Subsequently, proportional hazards assumption for different variables (Therneau, 1994) was examined by the scaled Schoenfeld residuals (Schoenfeld, 1982; R Development Core Team, 2014). In order to achieve the clinical application of survival prediction model, a prognostic nomogram was then constructed based on the outcomes of the multivariate Cox regression analysis (method = “enter”). Using the “rms,” “foreign,” and “survival” R packages, the nomogram was plotted based on the prognostic signature and six clinicopathology factors for the purpose of predicting 3-, and 5-OS of LGG. Furthermore, the concordance index (C-index) (Harrell et al., 1996) was employed to quantify predictive accuracies by using “survival” and “pec” package. Using the “timeROC” package of R, the time-dependent ROC curve was performed to estimate the prognostic power of the nomogram. To compare the accuracy and discrimination of different models (containing model 1: SI-risk signature; model 2: mRNAsi; model 3: corrected mRNAsi; model 4: six predominant clinic-pathological factors; model 5: model 4 + SI-risk signature), the net reclassification improvement (NRI) and the integrated discrimination improvement (IDI) were applied by using “survIDINRI” package (Pencina et al., 2008). Calibration curves were employed to evaluate the agreement between the observed and the predicted probability (3- and 5-years OS) in the nomogram. The bootstrap method with 1,000 resamples were utilized to evaluate both discrimination and calibration.

### External Validation of the Prognostic Signature

Another primary LGG of gene expression information and related predominant clinical and prognostic factors were downloaded from the CGGA platform^5^. A total of 353 samples were enrolled for external validation of the risk signature. The samples were uniformly divided into two distinct groups according to the same cutoff value (1.495), and the Kaplan–Meier analysis was employed to assess the high-risk and low-risk groups. Afterward, the ROC curve analysis was used to assess the discriminatory power of the risk score in the external validation set. Further, a heatmap was generated to show the gene expression distributions of signature genes in the CGGA database, and the risk plot showed the distribution of the LGG patients according to their individual risk score. Similarly, the C-index, the time-dependent ROC curves, and calibration curves (bootstrap method with 1,000 resamples) were compared to determine the performance of the risk signature.

### Cancer Cell Line Encyclopedia (CCLE) and Protein Expression Verification

The mRNA expression of seven genes profiled by RNA-Seq extracted from database available at The Cancer Cell Line Encyclopedia (CCLE)^6^ (Barretina et al., 2012). This portal covers genomic and expression data for more than 1000 cell lines from various tumors. The expression level of seven genes were analyzed in different types of cancer including LGG using CCLE. Cell lines of LGG were preliminary confirmed through six dedicated websites^7^ and only the consistent LGG cell lines be retained. In addition, the protein expression levels of the seven genes between glioma tissue and normal control were analyzed using Human Protein Atlas database^8^, and the data were visualized using immunohistochemistry staining.

### Statistical Analysis

The statistical analysis in our exploratory study was carried out using the R software (version 3.6.0)^9^. For differentially expressed gene selection, the Wilcoxon test was performed. The OCLR method was implemented with the “gelnet” package1 with default parameters (Sokolov et al., 2016). Pearson’s chi-square tests and Kruskal–Wallis tests were used to detect the variables difference. An analysis of the distinctness of survival between the two risk groups was illustrated by the Kaplan–Meier curve (Klein and Moeschberger, 1997) with the Wilcoxon logrank test using the R package KMsurv. The univariate Cox regression analysis and multivariate Cox regression analysis were performed to assess the association between the factors and OS (Therneau, 2015). A *p* < 0.05 was deemed as statistically significant.

## Results

### Data Processing

#### Identification of DEGs

**FIGURE 1:**
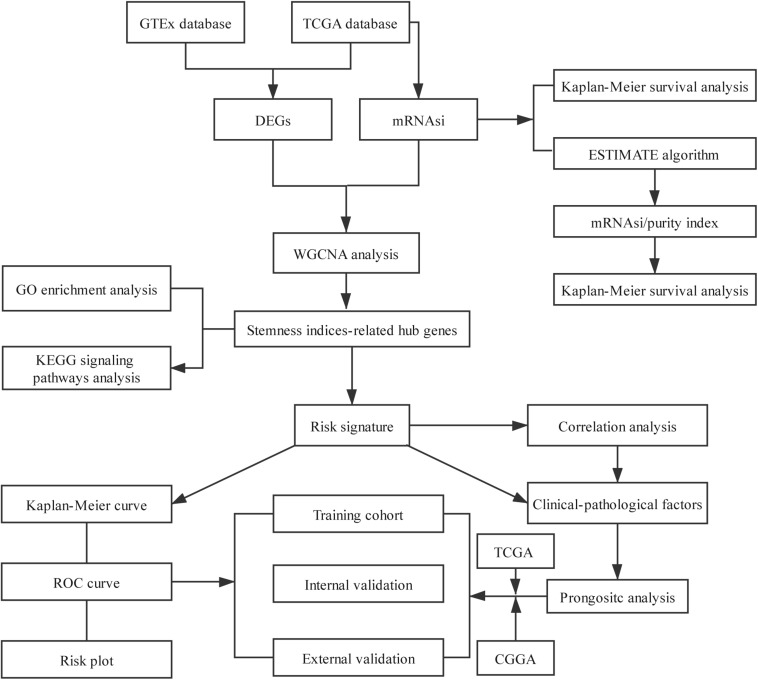
Flowchart presenting the process of establishing the stemness index-related signature.

The overview of the stemness index-related signature development and validation workflow is summarized in Figure 1. A total of 774 patients with primary LGG were enrolled in the generation of the stemness indices-associated risk signature, and the clinicopathological characteristics are listed in Table 1. The RNA-seq data (level 3) of 1,152 normal brain tissue samples and 529 LGG samples from GTEx projects and the TCGA were screened by the limma package. Before the identification of DEGs, the normalization and batch effect removal were tested. As illustrated in Supplementary Figures S1A,C, it performed well in normalization. Correspondingly, TCGA and GTEx samples separated obviously (Supplementary Figures S1B,D). Altogether, using the cutoff of significance of the absolute value of the log2-transformed fold change (FC) > 1 and the adjusted *P* value (adj.P) < 0.05, the differential expression analysis between 1,152 normal control samples and 529 LGG identified a cohort of 5,490 DEGs, of which 2,718 were upregulated and 2,772 were downregulated (Supplementary Figures S2A,B).

**TABLE 1:** Clinicopathological characteristics of primary LGG patients from the TCGA and CGGA databases.

Characteristic	Training cohort	Internal validation cohorts	External validation cohorts
	TCGA (*n* = 297)	TCGA (*n* = 124)	CGGA (*n* = 353)
**Age (Y)^a^**			
≤40	136 (46%)	63 (51%)	189 (54%)
¿40	161 (54%)	61 (49%)	164 (46%)
**Gender**			
Male	168 (57%)	65 (52%)	205 (58%)
Female	129 (43%)	59 (48%)	148 (42%)
**Grade**			
I	144 (52%)	54 (44%)	196 (56%)
II	153 (48%)	70 (56%)	157 (44%)
**Radiation**			
No	99 (37%)	53 (43%)	59 (17%)
Yes	198 (63%)	71 (57%)	294 (83%)
**Chemotherapy**			
No	133 (45%)	58 (47%)	147 (42%)
Yes	164 (55%)	66 (53%)	206 (58%)
**IDH^b^ Status**			
Wild-type	53 (18%)	26 (21%)	94 (27%)
Mutation	244 (82%)	98 (79%)	259 (73%)
**Risk score**			
Low risk	209 (70%)	80 (65%)	264 (75%)
High risk	88 (30%)	44 (35%)	89 (25%)

**^*a*^Age, Age at pathological diagnosis of glioma. ^*b*^IDH, Isocitrate dehydrogenase.**

#### mRNAsi Mining

Gene expression-based stemness indices for LGG were extracted by the one-class logistic regression machine learning algorithm (OCLR) (Malta et al., 2018). A cohort of LGG samples stratified by the mRNAsi, which is based on the stemness index model, were utilized for the integrative analyses.

### WGCNA: Construction the Correlation Matrix of mRNAsi and Module Eigengene Values

#### Data Acquisition

Using the TCGA database, a WGCNA network was constructed by the WGCNA package for the purpose of identifying stemness indices-related modules. The LGG transcriptome in the TCGA database was employed as the primary source for the analysis. Afterward, a global view of RNA-seq data analysis specific to LGG were provided by the WGCNA.

After data preprocessing, a correlation analysis of 5,490 DEGs was conducted, and the soft threshold power of β was 5 (scale-free *R*^2^ = 0.9) to assure a scale-free topology model (Supplementary Figure S3A). A total of 5,490 DEGs were screened for further analysis according to the exclusion criteria.

Next, a clustering analysis on this basis for LGG identified a total of eleven diverse modules (module size ≥ 50 and cut height ≥ 0.25) in the network (purple, turquoise, black, brown, magenta, green, red, yellow, blue, pink, and gray). Genes in the same color module demonstrated common gene expression patterns (Supplementary Figure S3B).

#### Identification of Modules Associated With Stemness Indexes of LGG

**FIGURE 2:**
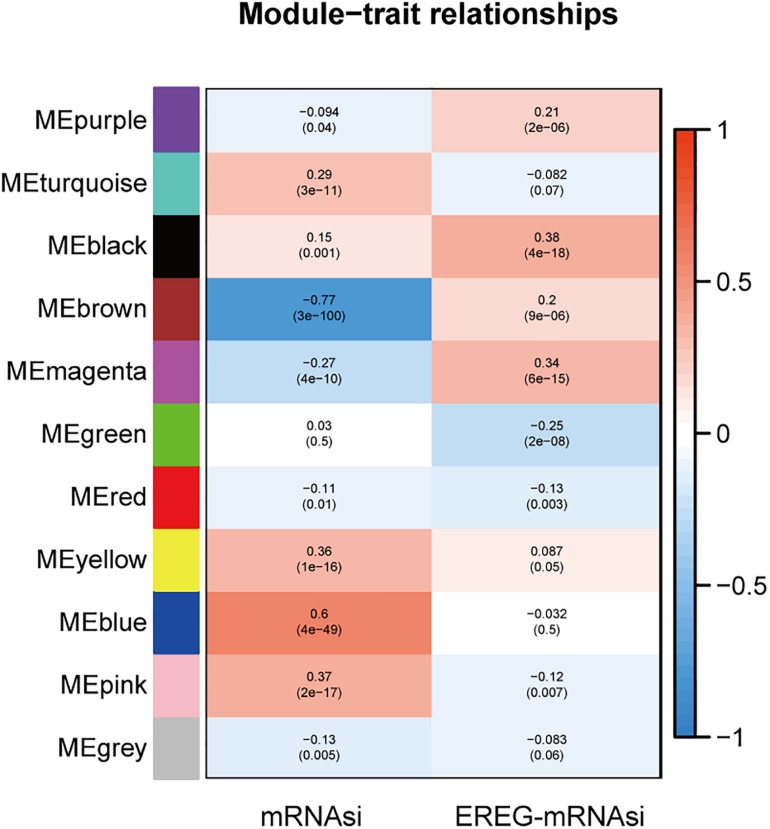
The analysis of the weighted gene correlation network analysis (WGCNA) modules showed that there were ten sets of genes (modules) significantly associated with mRNAsi. The purple, brown, magenta, red, and gray modules were correlated negatively with mRNAsi.

Fold enrichment > 1 and *p* < 0.05 was regarded as the statistical threshold of significance for mRNAsi associated modules selection. There were ten sets of genes (modules) identified that were significantly associated with mRNAsi. The purple, brown, magenta, red, and gray modules were correlated negatively with mRNAsi (ME_*purple*_:*r* = −0.094, *P* = 0.04, ME_brown_:*r* = −0.77, *P* = 3E^–100^, ME_magenta_:*r* = −0.27, *P* = 4E^–10^, ME_red_:*r* = −0.11, *P* = 0.01, ME_gr__a__y_:*r* = −0.13, *P* = 0.005). The turquoise, black, yellow, blue, and pink modules were correlated positively to mRNAsi (ME_turquoise_:*r* = 0.29, *P* = 3E^–11^, ME_black_:*r* = 0.15, *P* = 0.001, ME_yellow_:*r* = 0.36, *P* = E^–16^, ME_blue_:*r* = 0.6, *P* = 4E^–49^, ME_gr__a__y_:*r* = 0.37, *P* = 2E^–17)^ (Figure 2 and Supplementary Figure S3).

The module-trait relationships showed that the brown module was most significantly related to mRNAsi, with the highest correlation value (*r* = −0.77, *P* = 3E^–100^). Thus, the brown module was selected for subsequent analyses to explore key genes.

Based on the threshold limits (cor. gene GS > 0.5 and cor. gene MM > 0.8), 86 out of 748 hub genes were identified after selection in the brown module.

### Analysis and Functional Annotation of Key Genes in the Brown Module

#### Analysis of Key Genes in the Brown Module

The expression values of each key gene were retrieved from the normal control tissue and tumor tissue, which were visualized as heatmap (Supplementary Figure S4A). The heatmap showed that most of the key genes had median expression levels in tumor tissue, whereas CD74 Molecule (*CD74)*, major histocompatibility complex, class I, E (*HLA-E)*, major histocompatibility complex, class II, DR Alpha *(HLA-DRA)*, major histocompatibility complex, class II, DR Beta 1 *(HLA-DRB1)*, complement C1q B chain (*C1QB*), complement C1q A chain (*C1QA*), and complement C1q C chain (*C1QC*) exhibited the higher expression in samples from cancer patients. The correlation analyses between key genes were also visualized as a heatmap (Supplementary Figure S4B).

#### Functional Annotation of Genes Related to mRNAsi

**FIGURE 3:**
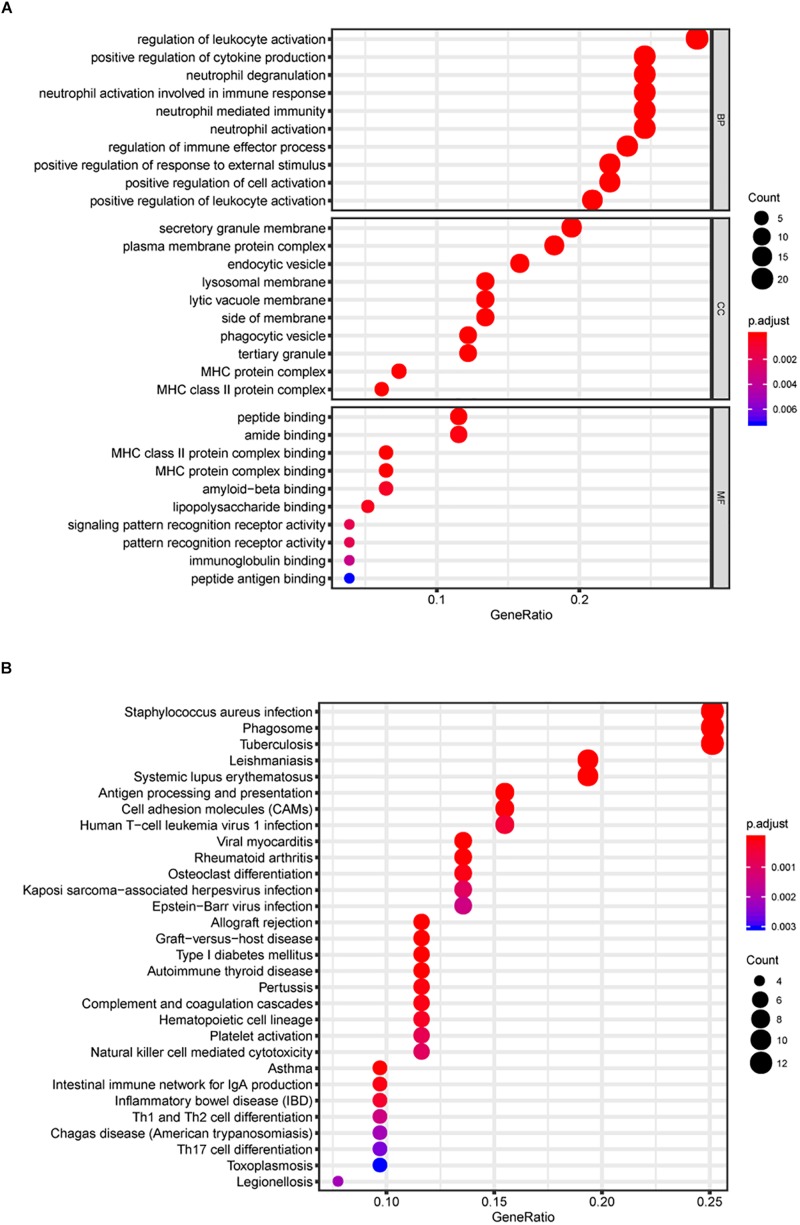
Gene Ontology (GO) and KEGG pathway analyses. In total, 30 GO biological process consisting of 10 biological processes (BP) terms, 10 cellular components (CC) terms, and 10 molecular functions (MF) terms were enriched **(A)**. Kyoto Encyclopedia of Genes and Genomes (KEGG) pathway analysis **(B)**.

The Gene ontology enrichment analysis was executed for further describing the function of the key genes. In total 30 GO biological processes consisting of 10 biological processes (BP) terms (regulation of leukocyte activation, etc.), 10 cellular components (CC) terms (secretory granule membrane, etc.), and 10 molecular functions (MF) terms (peptide binding, etc.) were enriched (Figure 3A).

In addition, KEGG signaling pathway analysis indicated that the key genes were significantly enriched in 30 pathways, and several pathways were immune-related, such as antigen processing and presentation and cell adhesion molecules (CAMs) (Figure 3B). The above results suggest the potential regulatory mechanism of mRNAsi-associated genes in the development of LGG.

#### Survival Analysis of mRNAsi

After calculating the mRNAsi for all LGG samples, a cohort of 447 patients with LGG were classified into either a high mRNAsi score group or a low mRNAsi score group, using the optimum cutoff value of 0.354. The survival curves showed that the OS values were significantly different between the two groups (*P* = 9.676E^–4^), based on Kaplan-Meier survival analysis (Supplementary Figure S5A).

Considering the interferences of tumor purity, the corrected mRNAsi (mRNAsi/tumor purity) was adopted. By applying ESTIMATE (Yoshihara et al., 2013), the tumor purity was calculated in any given LGG sample.

Similar results were also observed when the Kaplan-Meier survival analysis was applied to all the 463 samples based on corrected mRNAsi. There was a significant difference in OS between high mRNAsi score group and low mRNAsi score group (*P* = 5.019E^–4^) (Supplementary Figure S5B).

#### Identification of Key Prognostic Genes in Primary LGG

**FIGURE 4:**
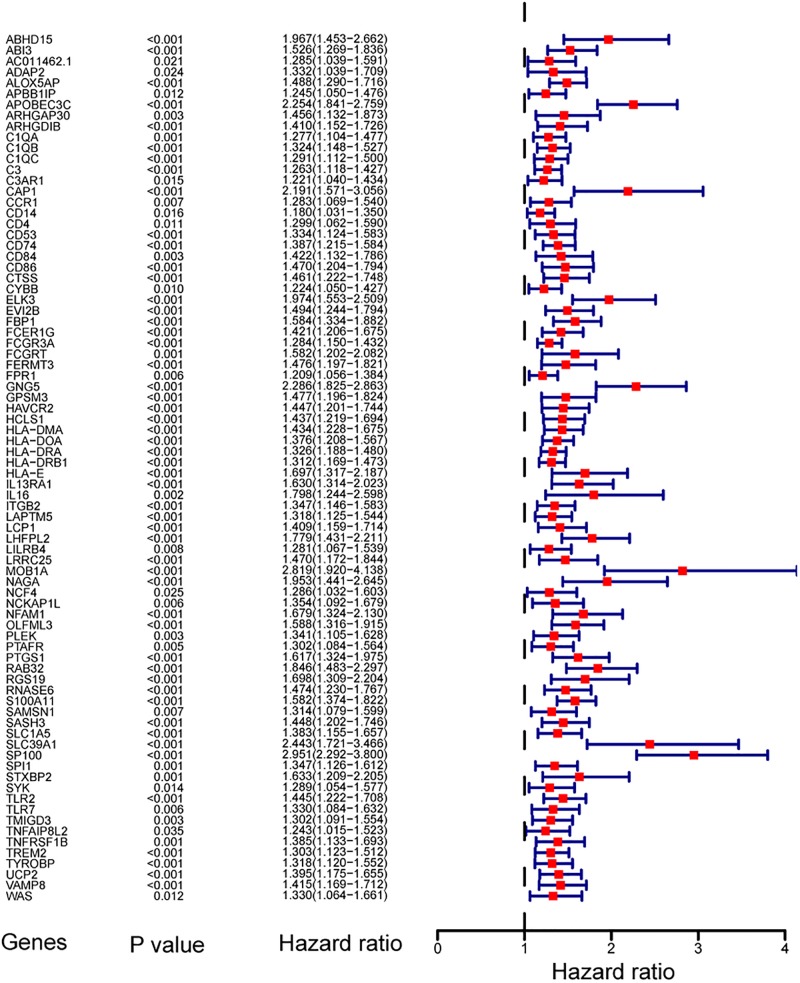
Forest plot showing the hazard ratios from the univariate Cox regression analysis.

To find out the prognostic value of stemness-index associated genes, 86 key genes were tested by univariate Cox regression analysis. It was found that 80 genes were significantly associated with OS in primary LGG. Surprisingly, all prognostic key genes were identified as risk factors (Figure 4).

#### Construction of Stemness-Index Associated Prognostic Signatures

Taking co-linearity into account, 80 key prognosis-related genes were subjected to LASSO Cox regression. A set of 11 key genes were then included in the subsequent analysis with non-zero regression coefficients. Next, 7 key genes were filtered and optimized for constructing a risk signature when implementing the stepwise multivariable Cox regression analysis (Table 2). The 7 key genes contained ArfGAP with dual PH domains 2 (*ADAP2*), arachidonate 5-lipoxygenase activating protein (*ALOX5AP*), apolipoprotein B mRNA editing enzyme catalytic subunit 3C (*APOBEC3C*), Fc fragment of IgG receptor and transporter (*FCGRT*), G protein subunit gamma 5 (*GNG5*), leucine rich repeat containing 25 (*LRRC25*), and SP100 nuclear antigen (*SP100*). Finally, a risk score formula was developed based on the seven key genes along with their individual coefficients and expression level, which was defined as follows: (−0.88603 × expression level of *ADAP2*) + (0.416964 × expression level of *ALOX- 5AP*) + (0.914674 × expression level of *APOBE- C3C*) + (−0.73585 × expression level of *FCGRT*) + (0.631697 × expression level of *GNG5*) + (−0.64501 × expression level of *LRRC25*) + (0.745358 × expression level of *SP100*).

**TABLE 2:** Results of the seven key genes in the multivariable Cox regression analysis.

Genes	Coef	HR	HR.95L	HR.95H	*P*-value
*ADAP2*	−0.886027724	0.412290235	0.22399138	0.758882942	0.004423098
*ALOX5AP*	0.416963664	1.517347377	1.10755058	2.07877013	0.009433367
*APOBEC3C*	0.914673555	2.495960324	1.783799483	3.492442955	9.47E-08
*FCGRT*	−0.735850888	0.479097627	0.294704586	0.778863129	0.002997479
*GNG5*	0.631697047	1.880799678	1.385572452	2.553029562	5.09E-05
*LRRC25*	−0.645008868	0.52465789	0.315056856	0.873702304	0.01318087
*SP100*	0.745358173	2.107196041	1.21584173	3.652017402	0.00789528

#### Evaluation of Survival Predicts the Accuracy of Seven-Gene-Based Signature

**FIGURE 5:**
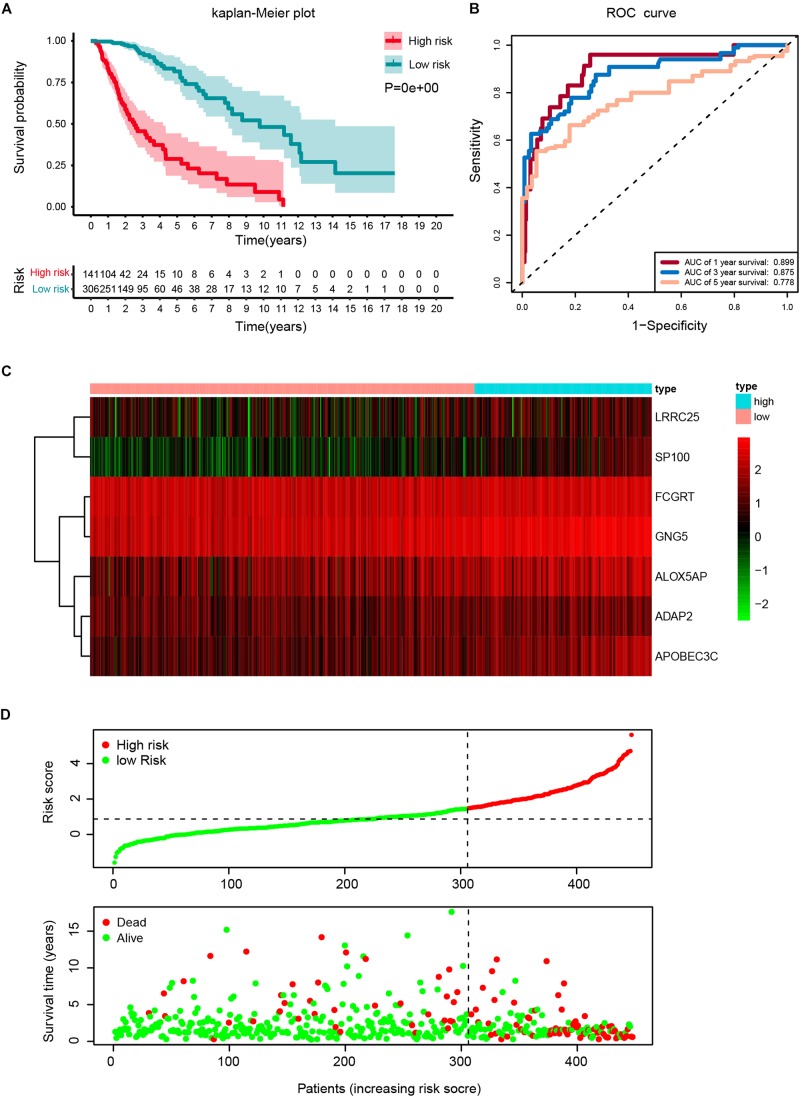
Construction of a risk score based on the seven stemness indices-related gene signature in the TCGA cohort. **(A)** Kaplan-Meier analysis of OS for low-risk and high-risk patients in the training cohort. Additionally, the table indicating the number at risk for each group at corresponding time points. **(B)** The time-dependent receiver operating characteristics (ROC) curve for 1-, 3-, and 5-year OS predictions for stemness-index related risk signature. **(C)** Heatmap showing the distribution of the expression of the seven genes of the stemness index in the TCGA cohort. **(D)** Risk plot presenting each point sorted based on risk score, representing one patient. Green and red points represent patients with low- and high-risk, respectively.

The robustness of the seven stemness-index associated genes was validated by evaluating the ability of stratifying the high-or low-risk group in TCGA datasets. Patients with primary LGG were dichotomized into high- (risk score ≥ 1.495) or low-risk group (risk score < 1.495) based on the optimal cutoff values. The Kaplan–Meier survival curve analysis showed that different risk groups by this risk scoring system were significantly linked with OS (Figure 5A). Next, the 1y-, 3y-, and 5y-AUC of the time-dependent ROC were 0.899, 0.875, and 0.778, respectively (Figure 5B), confirming the satisfactory prediction efficiency of the seven-gene stemness index-based signature in OS. Furthermore, as observed in the heatmap, *FCGRT* and *GNG5* had the highest expression levels, whereas *LRRC25*, *SP100*, *ALOX5AP*, *ADAP2*, and *APOBEC3C* exhibited low and medium expression levels (Figure 5C). Consecutively, the distribution of risk scores and survival status showed that patients with a risk score of 1.495 or higher generally had poorer survival when compared with another group (Figure 5D).

#### Prognostic Value of the Seven Gene-Based Signature

**FIGURE 6:**
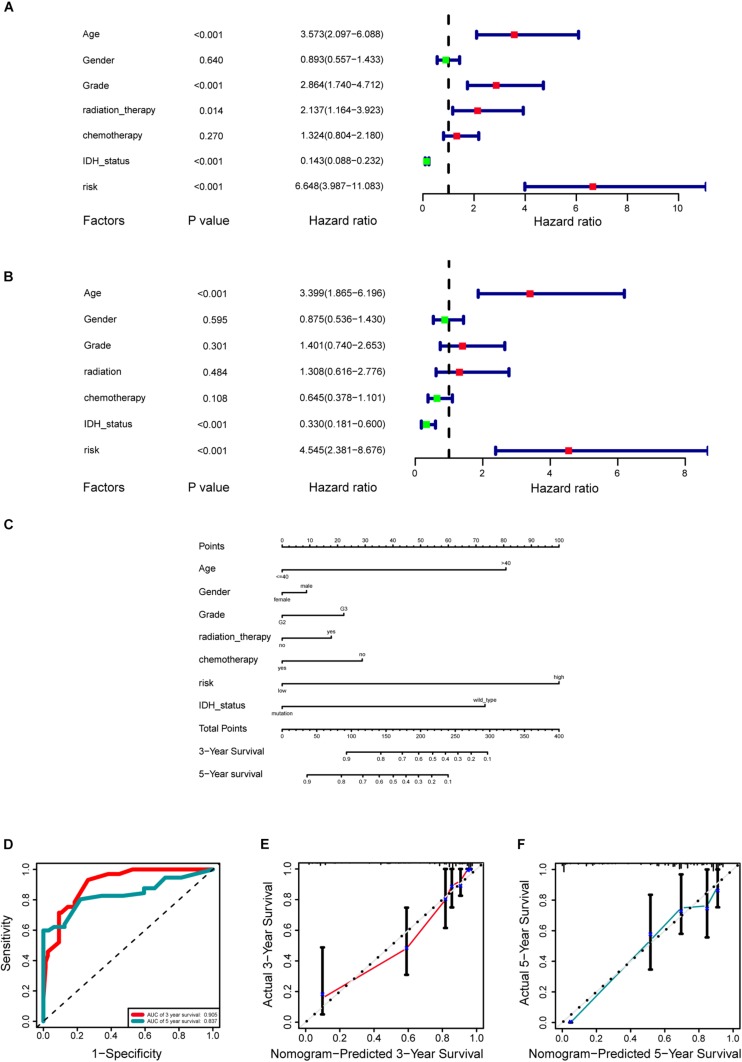
Development of a prognostic stemness indices-related gene signature for primary low-grade glioma (LGG). **(A)** Univariable Cox regression analysis for the training cohort. **(B)** Multivariable Cox regression analysis for the training cohort. **(C)** A nomogram including risk score and other clinical features for predicting 3- and 5-years overall survival (OS) of primary LGG. **(D)** Time-dependent receiver operating characteristics (ROC) curve analysis for 3- and 5-years OS predictions for the nomogram compared with actual observations. Calibration plot of nomogram for predicting probabilities of 3- **(E)** and 5-year **(F)** OS of primary LGG patients in the TCGA database.

A cohort of 421 patients with primary LGG in the TCGA database were classified into training set (*n* = 297) and internal validation set (*n* = 124) randomly at a ratio of 7:3. In consideration of the prognostic value of the stemness-index associated signature, the risk score was set as a potential factor and explored by the univariable and multivariable Cox regression analysis. The forest plot of the univariable Cox regression analysis, based on 6 clinicopathologic features showed that risk group (HR = 6.648, *p* < 0.001), age (HR = 3.573, *p* < 0.001), grade (HR = 2.864, *p* < 0.001), radiation therapy (HR = 2.137, *p* = 0.014), and IDH status (HR = 0.143, *p* < 0.001) were prognostic elements associated with OS (Figure 6A). Next, the results revealed that risk (HR = 4.545, *p* < 0.001), age (HR = 3.399, *p* < 0.001), and IDH status (HR = 0.330, *p* < 0.001) were statistically significant in multivariable Cox regression analyses (Figure 6B).

Based on the above results, the nomogram was established for predicting primary LGG 3- and 5-years survival, which integrated both the unique risk score and clinicopathologic variables (Figure 6C). The C-index of the nomogram was 0.8701 (95% CI; 0.8358–0.9044). The area under the curves (AUCs) of the 3- and 5-years OS predictions for the constructed nomogram were 0.905, and 0.837 in the training set, respectively (Figure 6D). Meanwhile, the calibration curves for this nomogram were developed and plotted in Figures 6E,F.

**FIGURE 7:**
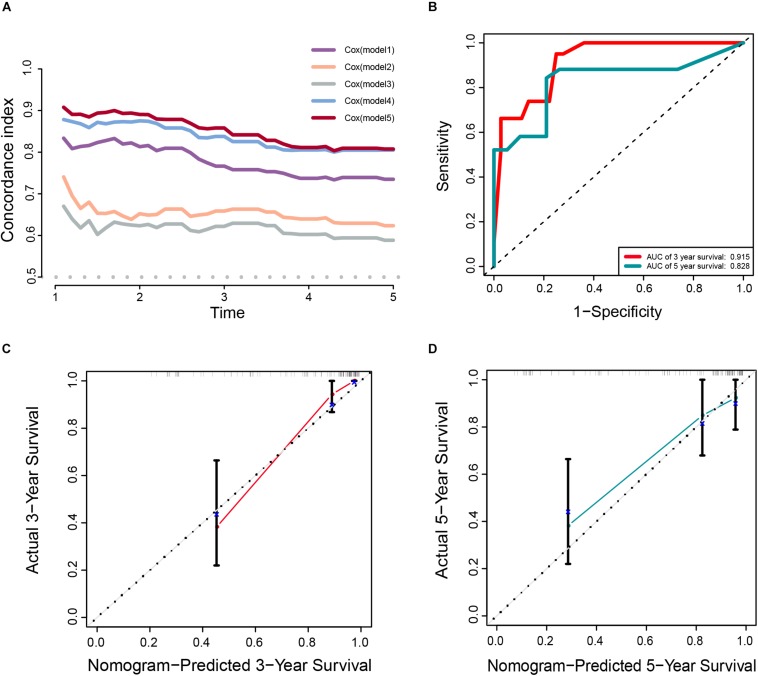
**(A)** The calculation of the C-indexes in five models. **(B–D)** Internal validation of a prognostic stemness index-related gene signature for primary LGG. **(B)** Time-dependent ROC curve of the seven-gene-based risk score for 3- and 5-year OS probability in the internal validation cohort. Calibration plot for internal validation of 3- **(C)** and 5-year **(D)** OS of primary LGG patients.

In addition, the comparison of the accuracy and discrimination in five models were conducted. The c-indexes of five models were 0.775, 0.658, 0.615, 0.852, and 0.870, respectively (Figure 7A). Moreover, as shown in Table 3, when defined the model 1 as the reference, the continuous NRI for the 1y-, 3y-follow ups were significant lower in mRNAsi group (model 2) with NRIs were -0.598 (*P* = 0.01) and -0.548 (*P* = 0.022). Correspondingly, the continuous NRI for the 1y-, 3y-follow ups were also significant lower in corrected mRNAsi group (model 3), with NRIs were -0.663 (*P* < 0.001) and -0.508 (*p* < 0.001). Conversely, the 1y-, 3y-NRI were significantly improved in model 4 and model 5 with NRIs were 0.458 (*P* = 0.016), 0.317 (*P* = 0.028), 0.708 (*P* < 0.001), and 0.433 (*P* < 0.001). Furthermore, the comparison between the model 4 and model 5 was also conducted. The 1y-, 3y-NRIs were also significant higher in model 5 (comprising all the seven factors in nomogram).

**TABLE 3:** Comprehensive comparison of the accuracy and discrimination in five models.

Index	Model 1 vs. Model 2	Model 1 vs. Model 3	Model 1 vs. Model 4	Model 1 vs. Model 5	Model4 vs. Model 5
IDI (1 year)	−0.037 (*p* = 0.274)	−0.063 (*p* = 0.02)	0.084 (*p* = 0.002)	0.108 (*p* < 0.001)	0.025 (*p* = 0.186)
Continuous NRI (1 year)	−0.598 (*p* = 0.010)	−0.663 (*p* < 0.001)	0.458 (*p* = 0.016)	0.708 (*p* < 0.001)	0.422 (*p* = 0.032)
IDI (3 year)	−0.146 (*p* = 0.040)	−0.178 (*p* = 0.006)	0.165 (*p* = 0.014)	0.214 (*p* < 0.001)	0.049 (*p* = 0.102)
Continuous NRI (3 year)	−0.548 (*p* = 0.022)	−0.508 (*p* < 0.001)	0.317 (*p* = 0.028)	0.433 (*p* < 0.001)	0.508 (*p* = 0.032)
IDI (5 year)	−0.189 (*p* = 0.044)	−0.211 (*p* = 0.056)	0.122 (*p* = 0.158)	0.177 (*p* = 0.018)	0.055 (*p* = 0.292)
Continuous NRI (5 year)	−0.530 (*p* = 0.078)	−0.366 (*p* = 0.058)	0.157 (*p* = 0.274)	0.410 (*p* = 0.036)	0.398 (*p* = 0.106)

**Model 1: only the SI−risk signature was enrolled in the prognostic factor; Model 2: mRNAsi was enrolled in the prognostic factor; Model 3: corrected mRNAsi was enrolled in the prognostic factor; Model 4: age, gender, grade, radiation therapy, chemotherapy, and IDH status were enrolled in the prognostic factors; Model 5: age, gender, grade, radiation therapy, chemotherapy, IDH status, and risk group were enrolled in the prognostic factors. NRI, net reclassification improvement; IDI, integrated discrimination improvement.**

Moreover, the 3y-, 5y IDI were significantly decreased in model 2 (IDI = -0.146 and -0.189). The 1y-, 3y-IDI were significantly decreased in model 3 (IDI = -0.063 and -0.178) with borderline significance in 5y-IDI (*P* = 0.056). Conversely, the 1y-, 3y-IDI were significant higher in model 4 (IDI = 0.084 and 0.165). Interestingly, 1y-, 3y-, 5y-IDI were all significant improved in model 5. In terms of the comparison of IDI between model 4 and model 5, despite the IDI were all improved, however, the *P* values could not reach the levels of significance.

#### Internal Validation of Seven-Gene Stemness-Index Associated Prognostic Signature

Meanwhile, the clinical predictive model was evaluated in an internal validation set. The C-index was 0.8474 (95% CI; 0.7081–0.7971), the area under the curves (AUC) for 3 and 5-years-survival were 0.915 and 0.828, respectively (Figure 7B). Taking the calibration curves for the nomogram-probability of 3-years survival (Figure 7C) and 5-years survival (Figure 7D) together, the seven-gene signature was capable of predicting the OS of primary LGG patients with high efficiency.

#### Development and External Validation of the Prognostic Signature

**FIGURE 8:**
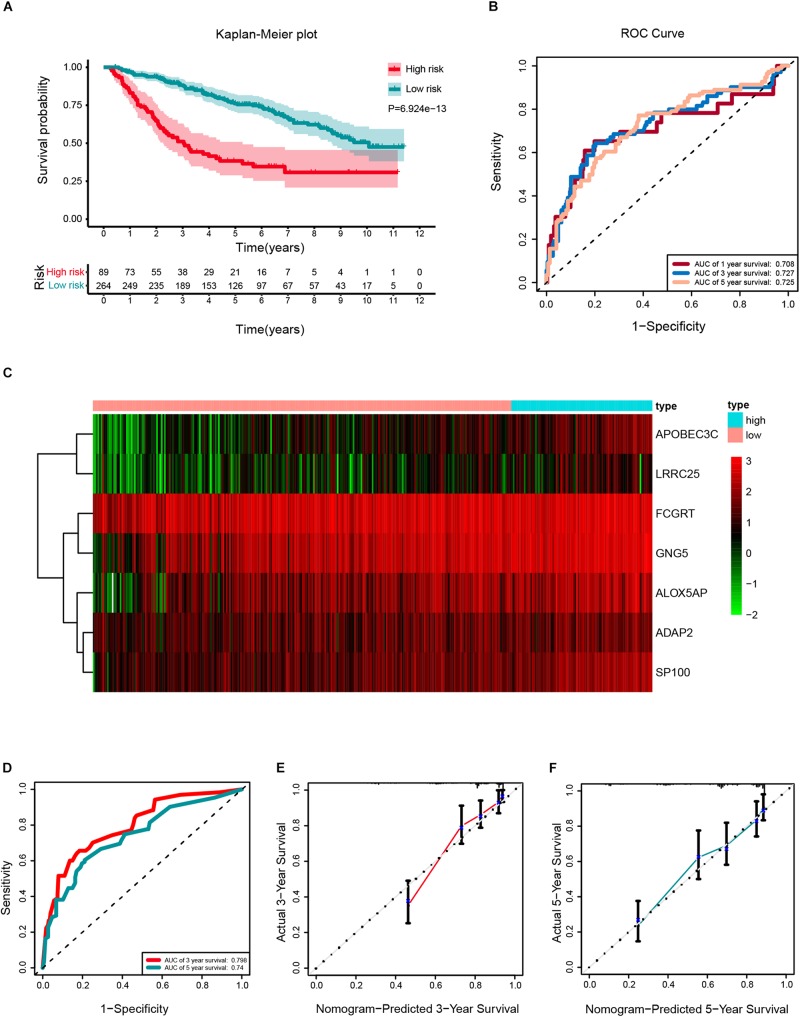
External validation of a prognostic stemness index-related gene signature for primary low-grade glioma (LGG). **(A)** Kaplan-Meier analysis of OS for low-risk and high-risk patients in the external validation cohort. Additionally, the table indicating the number at risk for each group at corresponding time points. **(B)** The time-dependent receiver operating characteristics (ROC) curve for 1-, 3-, and 5-years OS predictions for the nomogram compared with actual observations. **(C)** The heatmap shows the expression of the seven genes between two risk groups in the CGGA cohort. **(D)** Time-dependent ROC for 3- and 5-years OS predictions for the nomogram compared with actual observations. Calibration plot of nomogram for predicting probabilities of 3- **(E)** and 5-year **(F)** OS of primary LGG patients in the CGGA database.

According to the same cut-off value, the external validation set of 353 patients in the CGGA platform was employed and divided into high-risk cohort *(n* = 89) and low-risk cohort (*n* = 264). Similar procedures were conducted to assess the performance of the stemness-index associated signature. Using the Kaplan-Meier curve analysis, the high-risk cohort also showed a significantly poorer prognosis than the low-risk cohort (*P* = 6.924E^–13^) (Figure 8A). The 1y-, 3y-, 5y-AUC in the external validation set were 0.708, 0.727, and 0.725, respectively (Figure 8B). In accordance with the risk plot in the TCGA database, In accordance with the risk plot in the TCGA database, there was an inverse relationship between risk score and survival (Figure 8C). Subsequently, the AUCs for 3- and 5-years OS were 0.798, and 0.74, respectively (Figure 8D). The C-index in the external validation set was 0.7526. The calibration curves for the nomogram 3- and 5-year survival probabilities are shown in Figures 8E,F, respectively.

#### Evaluation of the Correlation Between Clinical Parameters and Signature

The relationship between the clinicopathological features (age, gender, grade, radiotherapy, chemotherapy, and IDH mutation status) and the seven-gene-based signature was explored. Older patients, patients of grade III, and IDH wild type tended to have higher risk scores than the younger, grade II, and the IDH mutant type patients, respectively in the TCGA database (Supplementary Figure S6A). As for the CGGA database, the risk scores of patients with IDH1 mutant type, and grade II were lower than IDH1 wild type, and grade III, respectively (Supplementary Figure S6B).

#### Expression Analysis of Seven Genes From Cancer Cell Line Encyclopedia (CCLE) and Human Protein Atlas Database

To validate the mRNA expression of seven genes, the expression levels of *ADAP2*, *ALOX5AP*, *APOBEC3C*, *FCGRT*, *GNG5*, *LRRC25*, and *SP100* in various human tumors and 14 LGG cell lines from the CCLE were determined (Supplementary Figure S7 and Table 4). As shown in Supplementary Figure S7, the mRNA expression of *APOBEC3C*, *FCGRT*, *GNG5*, and *SP100* was elevated in glioma, whereas the expression of *ADAP2*, *ALOX5AP*, and *LRRC25* was low. To further explore the expression patterns of the seven genes in tissue level, the Human Protein Atlas database was employed to analyze the differential expression between glioma tissue and normal control, and the protein expression was evaluated using immunohistochemistry data as shown in Supplementary Figure S8. Consistent with the RNA-seq data, the protein expression levels of *FCGRT*, and *GNG5* were upregulated in tumor tissues when compared with the normal controls.

**TABLE 4:** List the expression of the seven genes in 14 LGG cell lines.

Gene	*ADAP2*	*ALOX5AP*	*APOBEC3C*	*FCGRT*	*GNG5*	*LRRC25*	*SP100*	*ACTB*	RRID
**Gene expression (TPM)**									
H4	–3.234	–2.279	4.977	0.095	4.544	–4.032	3.775	10.664	CVCL_1239
HS683	–2.058	–0.673	4.300	2.101	6.059	–6.675	3.185	11.276	CVCL_0844
KG1C	–1.336	–1.609	6.160	0.334	5.218	–5.833	3.823	9.971	CVCL_2971
LN215	–3.879	–1.422	4.532	3.533	6.074	–7.049	4.243	10.537	CVCL_3954
LN235	–5.352	–1.489	4.216	3.967	6.028	–13.000	2.936	11.236	CVCL_3957
LN319	–4.063	–4.098	4.875	4.220	6.443	–13.000	1.443	9.752	CVCL_3958
LNZ308	–4.406	–3.171	2.872	0.317	7.113	–6.339	2.853	11.534	CVCL_0394
NMCG1	–3.648	–4.420	5.862	3.947	5.529	–8.266	2.392	11.488	CVCL_1608
SF268	–4.211	–1.512	0.554	3.739	6.236	–8.445	2.833	11.989	CVCL_1689
SNU738	–4.872	0.014	3.532	–3.016	6.417	–13.000	1.792	11.811	CVCL_5087
SW1088	–5.636	–2.916	5.441	1.140	6.274	–13.000	3.756	11.187	CVCL_1715
SW1783	–3.378	–1.839	4.975	2.773	5.975	–13.000	2.574	11.152	CVCL_1722
TM31	–2.523	–1.341	4.058	3.116	6.403	–8.164	1.793	10.397	CVCL_6735
U178	–3.724	–0.034	5.055	–3.648	5.367	–6.019	3.714	11.720	CVCL_A758

## Discussion

In previous studies, the risk stratification of the stemness index has been investigated in pan-cancer cohorts. However, the comprehensive prognostic value of the stemness index has not been exploited in LGG. In addition, the function annotation of the stemness index-associated genes and the prognostic value of the risk signature have not been investigated. In the current study, significant differences were found in survival between low- and high mRNAsi (mRNAsi/purity) score groups in the Kaplan Meier curve. Moreover, the detail of stemness indices-related modules and genes were identified after the application of WGCNA. A total of 86 key genes were screened according to the threshold limits, which were most significantly correlated with stemness-index. Next, for the enrichment analysis of the brown module, GO terms consisting of “regulation of leukocyte activation,” “positive regulation of cytokine production,” and “neutrophil degranulation” were ranked at the top of the list. In addition, KEGG pathway results such as CAMs, natural killer cell mediated cytotoxicity, and antigen processing and presentation were also obtained. Next, after the application of univariate Cox regression analysis, LASSO Cox regression model, and multiCox analysis, seven key genes (*ADAP2*, *ALOX5AP*, *APOBEC3C*, *FCGRT*, *GNG5*, *LRRC25*, and *SP100*) were enrolled as vital elements in stemness index-related signature. Furthermore, age, grade, radiotherapy, IDH status, and risk group were significantly associated with OS in the univariable Cox regression analysis; however, only age, IDH status, and risk group were significantly correlated with OS for primary LGG patients by applying the multivariate Cox regression analysis.

In the first part, it was found that the mRNAsi was significantly associated with OS in primary LGG, which was consistent with a previous study in pan-cancer cohorts (Malta et al., 2018). However, it should be noticed that the population of bulky tumor includes tumor cells, immune cells, and stromal cells. Taking the tumor purity into account may accurately reflect the actual stemness characteristic in tumor parenchyma. Moreover, ESTIMATE is one of the most common algorithms for quantifying tumor purity and composition of stromal and immune cells. Hence, the concept of the corrected mRNAsi (mRNAsi/tumor purity) was adopted to reduce the interference of non-tumor tissue (Malta et al., 2018; Lian et al., 2019; Pan et al., 2019). Of note, after employing the survival analysis, the significant survival difference in OS was still observed between high- and low- score groups based on the corrected mRNAsi (mRNAsi/tumor purity), which was consistent with the results from a previous study of bladder cancer (Pan et al., 2019). Additionally, the comparisons of the accuracy and discrimination among three models (model 1, model 2, and model 3) were conducted. Interestingly, the constructed risk signature in current study was superior to the mRNAsi and corrected mRNAsi in predicting the overall survival of LGG. To our knowledge, there is no previous study investigating the improvements of the accuracy and discrimination between mRNAsi and corrected mRNAsi. Further pan-cancer analyses are warranted.

To gain insights into the biological functions of key genes in WGCNA, it was found that the key genes were mainly enriched in infiltration, inflammation, and immune-related pathways, which were critically involved in the initiation and progression of glioma (Balkwill and Mantovani, 2001; Shacter and Weitzman, 2002; Mantovani et al., 2008; Michelson et al., 2016; Mostofa et al., 2017). Several studies have explored the prognostic value of host inflammatory cells, such as neutrophils in glioma. The role of neutrophils in glioma has two sides, mainly depending on the maturation and activation state. For example, the series of infiltrating neutrophils have the ability of contributing to glioma infiltration and pro-tumoral activity by secreting elastase (Iwatsuki et al., 2000). Circulating neutrophil-induced immunosuppression can promote tumor growth by secretion of arginase I (Sippel et al., 2011). On the other side, it has been found that the activation of neutrophils have an anti-tumor effect through antibody-dependent cellular cytotoxicity (Hafeman and Lucas, 1979; Fanger et al., 1989). Apart from making use of the migration of neutrophils, anti-cancer drugs can be delivered to the inflamed brain in glioma patients after surgery, which may reduce the recurrence of glioma (Xue et al., 2017). Moreover, recent evidence has revealed that the potential role of phagosomes in tumorigenesis via different mechanisms including its engagement in the autophagy pathway (Kim and Overholtzer, 2013).

Several studies have focused on the role that stemness features play in survival outcomes in human cancers. Similar to our study, using 763 primary medulloblastoma patients from the Gene Expression Omnibus (GEO) datasets, Lian and colleagues identified and validated a stemness-related gene expression signature to effectively stratify patients with Sonic hedgehog medulloblastoma into different OS groups (HR = 1.80, 95% confidence interval: 1.45–2.24, *P* = 1.10E^–07^) (Lian et al., 2019). In terms of LGGs, age (≤40 years vs. > 40 years), tumor grade (II vs. III), and IDH status (wild-type vs. mutation) are well-established and widespread prognostic biomarkers in clinical practice (Ricard et al., 2012; Cancer Genome Atlas Research et al., 2015; Zeng et al., 2018; National Comprehensive Cancer Network, 2019).

In the present study, a seven-gene signature based on the mRNAsi was built to predict the prognosis of LGG. After the univariate and multivariate analysis, the stemness index-related gene signature, age, and IDH status were identified as independent prognostic markers for predicting OS in primary LGG patients. To our surprise, receiving radiation therapy and chemotherapy or not was not associated with OS in the multivariate analysis. The reason might be the undefined and inconsistency treatment protocols, including the duration of treatment, cycles of chemotherapy, total or fraction radiation dose, and combined treatment regimens. Moreover, numerous clinical trials have provided evidence for the adoption of chemotherapy and radiotherapy in gliomas and confirmed the OS benefit in adjuvant therapy. The Radiation therapy oncology group (RTOG) 9802 trial showed that radiotherapy combined with adjuvant procarbazine, 3 CCNU, and vincristine (PCV) chemotherapy substantially improves the median OS from 7.8 to 13.3 years (HR = 0.59; *P* = 0.002) in low-grade glioma patients older than 40 years or who did not undergo total tumor resection (van den Bent, 2014). Additionally, despite the six clinic-pathological factors comprised model performed fairly in predicting OS, however, integrating the risk signature further improve the c-index as well as the significant enhancements of 1y- and 3y-NRI. Thus, new prospective studies are necessary to further verify the prognostic value of the stemness index-associated risk signature in primary LGG patients who receive a combined standard approach of surgery, radiotherapy, and chemotherapy.

Among the seven genes, *ALOX5AP*, *APOBEC3C*, *GNG5*, and *SP100* were identified as risk-associated genes, whereas *ADAP2*, *FCGRT*, and *LRRC25* were confirmed as protective genes. Regarding the risk-associated genes, *APOBEC3C* was discovered as a vital member of the APOBEC family that encodes the APOBEC3C (apolipoprotein B mRNA editing enzyme catalytic subunit 3C, or A3C), clustered in the human chromosome 22 (Jarmuz et al., 2002). Some investigations have shown that the expression of *APOBEC3C* played a positive role in the invasiveness and prognosis of breast cancer (Zhang et al., 2015; Wang et al., 2019), hepatocellular carcinoma (Yang et al., 2015), and prostate cancer (Kawahara et al., 2019). Taking into account investigations on the role of GNG5 in carcinogenesis, Orchel et al. (2012) found that GNG5 may play a vital role in pathogenesis or progression of endometrial cancer. In addition, it has been revealed that GNG5 involved in PI3K-AKT and Wnt signaling pathway, and associated with reduced E-cadherin expression in invasive breast cancer (Alsaleem et al., 2019). *ALOX5AP* is one of the essential genes in the production of leukotrienes from arachidonic acid via encoding the ALOX5AP. Consistent with our results, Wu et al. (2018) found that high expression of *ALOX5AP* is associated with poor survival outcome in esophageal carcinoma. Additionally, ALOX5AP also involved in a risk model to serve as a prediction of osteosarcoma metastasis (Dong et al., 2019). It is known that the nuclear autoantigen SP100 participates in various biological processes, such as cellular gene expression, differentiation, and cell growth (Everett et al., 2006). It was found that high expression of *SP100* was associated with poor cell differentiation in laryngeal cancer (Li et al., 2010). Moreover, the expression of SP100 could regulate the transcriptional activity of ETS1 and further influence the cell invasion in breast cancer (Yordy et al., 2004). Regarding the role of *SP100* in glioma, previous study revealed that *SP100* was overexpressed in glioblastoma cells and involved in the regulation of glioblastoma cell proliferation and migration (Held-Feindt et al., 2011). With regards to the protective genes of *FCGRT*, it has been found to be responsible for encoding neonatal Fc receptor (FcRn), which participates in the transport and homeostasis of immunoglobulin as well as anti-tumor immunity (Roopenian and Akilesh, 2007; Ward and Ober, 2009). The expression of FcRn in immune cells, particularly in antigen presenting cells, is associated with its involvement in antigen presentation and cross-presentation that contributes to its shape anti-tumor properties. Studies showed that FcRn-expressed dendritic cells (DCs) are critical for the number and activation of CD8 + T-cells and are associated with prognosis in colorectal carcinoma (Baker et al., 2013). The downregulation of FcRn is correlated with reducing maturation and activation of natural killer cells that in turn increase lung metastasis in an FcRn-depleted environment in mice (Castaneda et al., 2018). The protein encoded by ADAP2 is a GTPase-activating protein and increases the stability of microtubules. The investigation about the role of *ADAP2* in solid tumor is rare. Only one study found the expression of ADAP2 was markedly decreased in *in vivo* tumors without further validation about the function or mechanism (Laukkanen et al., 2015). Correspondingly, the prognostic value of *LRRC25* has not been investigated in solid tumors. Hoffman et al. found that the expression of *LRRC25* was significantly associated with the risk of breast cancer (Hoffman et al., 2017). Further investigations are warranted to explore the mechanisms of *LRRC25* in glioma.

Several limitations should be noticed in the current study. First, the stemness index-related signature and the nomogram developed were able to accurately predict survival outcome in primary LGG. Nonetheless, the validation in cellular experiments, and animal and tissue models warrants further investigation. Second, due to an absence of 1p19q characterization in the TCGA datasets, the status of 1p19q co-deletion was not investigated by the univariate and multivariate Cox regression analysis and was not employed for the establishment of prognostic nomogram. Third, considering a lack of standard treatment strategies in the TCGA and CGGA databases, the effectiveness of the seven-gene signature in primary LGG patients who received standard treatment needs to be further verified in well-designed prospective clinical investigations.

## Conclusion

Our study identified a novel gene signature based on seven genes relevant to the stemness index and developed a prognostic nomogram composed of the gene signature and clinical prognostic factors that effectively predict overall survival in primary LGG patients. *ALOX5AP, APOBEC3C*, *GNG5*, *SP100, ADAP2*, *FCGRT*, and *LRRC25* might be candidate prognostic biomarkers in primary LGG.

## Data Availability Statement

Publicly available datasets were analyzed in this study. The RNA-seq data (level 3) and clinical information of LGG samples can be found in the UCSC Xena browser (http://xena.ucsc.edu/), and the CGGA database (http://www.cgga.org.cn). The mRNA expression-based stemness index (mRNAsi) was provided by Tathiane M. Malta, et al.

## Ethics Statement

All the information of patients was obtained from the Chinese Glioma Genome Atlas (CGGA), and The Cancer Genome Atlas (TCGA). All the patients and treatments complied with the principles laid down in the Declaration of Helsinki of 1964 and its later amendments or comparable ethical standards.

## Author Contributions

MZ analyzed the data. MZ, XC, and JH contributed materials and analysis tools. XW prepared figures and tables. MZ, XC, and XW authored or reviewed drafts of the manuscript. FG and JH conceived and designed the study. FG revised the manuscript.

## Conflict of Interest

The authors declare that the research was conducted in the absence of any commercial or financial relationships that could be construed as a potential conflict of interest.

## Footnotes

1 https://xena.ucsc.edu/

2 https://cran.r-project.org/web/packages/WGCNA/index.html

3 https://bioinformatics.mdanderson.org/estimate/

4 https://cran.r-project.org/web/packages/caret

5 http://cgga.org.cn/

6 https://portals.broadinstitute.org/ccle

7 https://web.expasy.org/cellosaurus/, https://www.atcc.org/, https://www.pheculturecollections.org.uk/products/celllines/generalcell/search.jsp, http://igrcid.ibms.sinica.edu.tw, https://cansarblack.icr.ac.uk/, https://www.dsmz.de/

8 http://www.proteinatlas.org/

9 https://www.r-project.org/

## Supplementary Material

FIGURE S1 The normalization and batch effect removal from TCGA and GTEx datasets. **(A)** Box plots illustrated the data distributions from TCGA and GTEx datasets before normalization. **(B)** PCA plot illustrated the cluster of the samples from TCGA and GTEx datasets before batch effect removal. **(C)** Box plots illustrated the data distributions from TCGA and GTEx datasets after normalization. **(D)** PCA plot illustrated the cluster of the samples from TCGA and GTEx datasets after batch effect removal.

FIGURE S2 **(A)** Heatmaps showing that the 5,490 differentially expressed genes (DEGs) can effectively distinguish tumors from non-tumor tissues after integrated analysis. **(B)** Volcano plot presenting DEGs between LGG and non-tumor tissues. Red dots, and green dots represent up-regulated genes, and down-regulated genes, respectively.

FIGURE S3 Weighted gene correlation network analysis for building stemness-index associated preserved Modules. **(A)** Determination of soft threshold for adjacency matrix, and plots of mean connectivity versus soft threshold. **(B)** Clustering results of WGCNA modules. The horizontal axis indicates modules with different colors.

FIGURE S4 Analysis of key genes in the module brown. **(A)** The heatmap showing that the differentially expressed levels of the key genes between the normal control tissue and tumor tissue. **(B)** The heatmap of the correlation analysis among key genes.

FIGURE S5 **(A)** Kaplan-Meier survival analysis of mRNAsi. **(B)** Kaplan-Meier survival analysis of corrected mRNAsi. Additionally, the table indicating the number at risk for each group at corresponding time points.

FIGURE S6 Association between risk score and clinical-pathological parameters. Association between risk score and age, gender, grade, radiotherapy, chemotherapy, and IDH mutation status of primary LGG patients in TCGA cohort **(A)**, in CGGA cohort **(B)**.

FIGURE S7 The mRNA expression level of *ADAP2* **(A)**, *ALOX5AP* **(B)**, *APOBEC3C* **(C)**, *FCGRT* **(D)**, *GNG5* **(E)**, *LRRC25* **(F)**, and *SP100* **(G)** in different types of human cancers.

FIGURE S8 The protein expression level of immunohistochemistry (IHC) images collected from the Human Protein Atlas database of the risk genes between glioma tissue and normal control (ADAP2 was not available).
