# Supplementary figures and images for: Prognostic Value of a Stemness Index-Associated Signature in Primary Lower-Grade Glioma
Source: Front Genet. 2020 May 5;11:441. doi: 10.3389/fgene.2020.00441 (PMC7216823; doi:10.3389/fgene.2020.00441)

**A**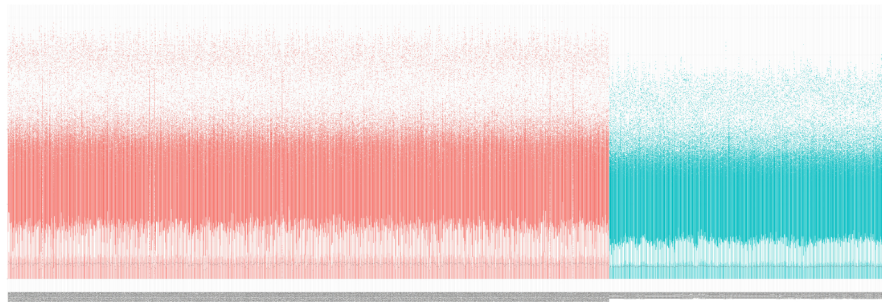**B**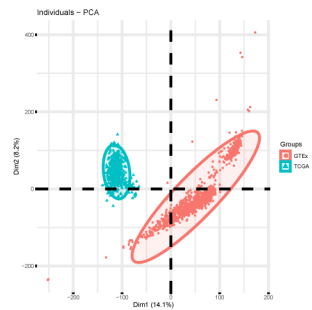**C**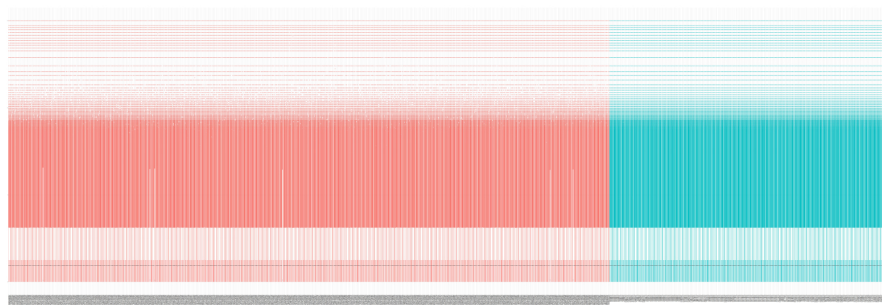**D**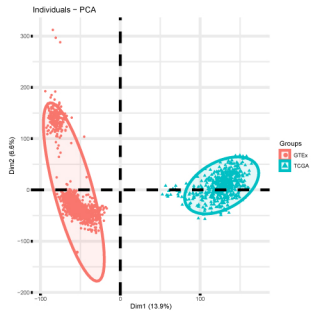

Supplement: FIGURE S1 — The normalization and batch effect removal from TCGA and GTEx datasets. (A) Box plots illustrated the data distributions from TCGA and GTEx datasets before normalization. (B) PCA plot illustrated the cluster of the samples from TCGA and GTEx datasets before batch effect removal. (C) Box plots illustrated the data distributions from TCGA and GTEx datasets after normalization. (D) PCA plot illustrated the cluster of the samples from TCGA and GTEx datasets after batch effect removal. [file Image_1.PDF]

**A**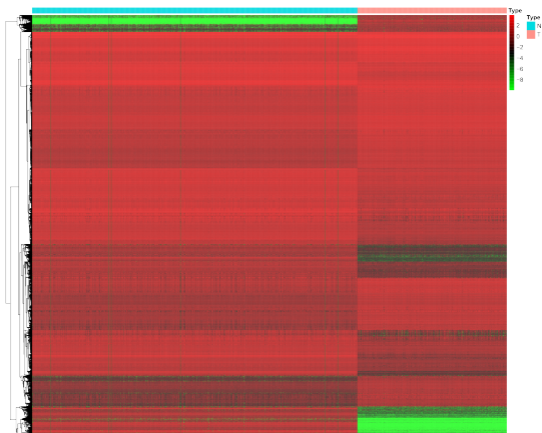**B****Volcano**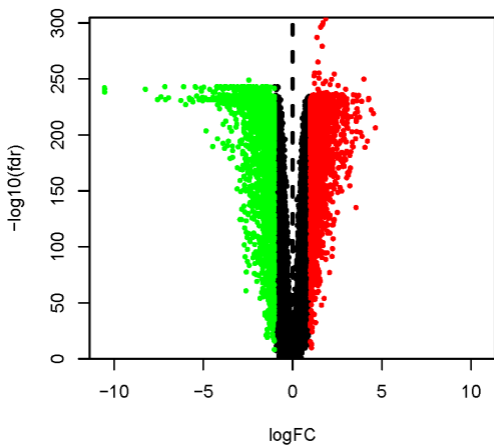

Supplement: FIGURE S2 — (A) Heatmaps showing that the 5,490 differentially expressed genes (DEGs) can effectively distinguish tumors from non-tumor tissues after integrated analysis. (B) Volcano plot presenting DEGs between LGG and non-tumor tissues. Red dots, and green dots represent up-regulated genes, and down-regulated genes, respectively. [file Image_2.PDF]

**A**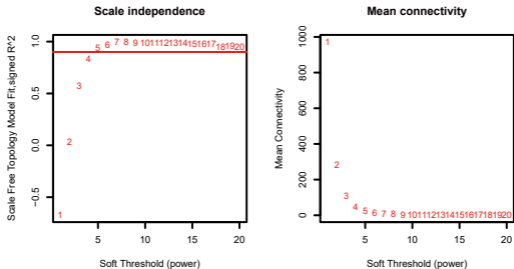**B**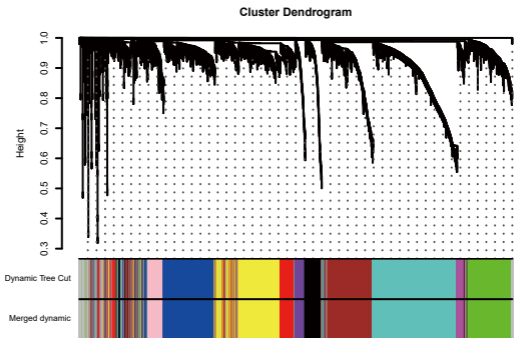

Supplement: FIGURE S3 — Weighted gene correlation network analysis for building stemness-index associated preserved Modules. (A) Determination of soft threshold for adjacency matrix, and plots of mean connectivity versus soft threshold. (B) Clustering results of WGCNA modules. The horizontal axis indicates modules with different colors. [file Image_3.PDF]

**A**

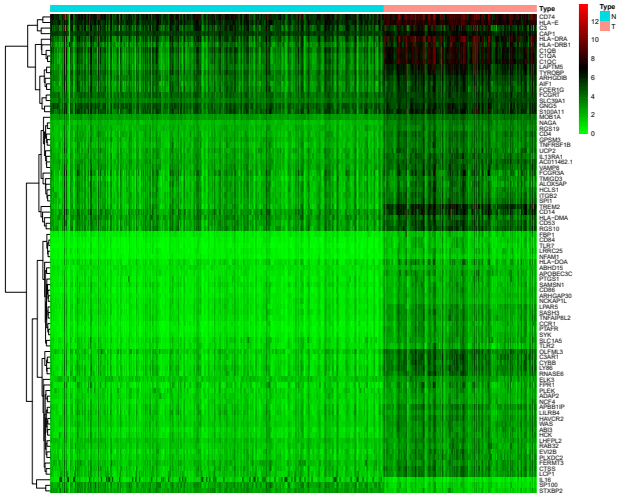

B

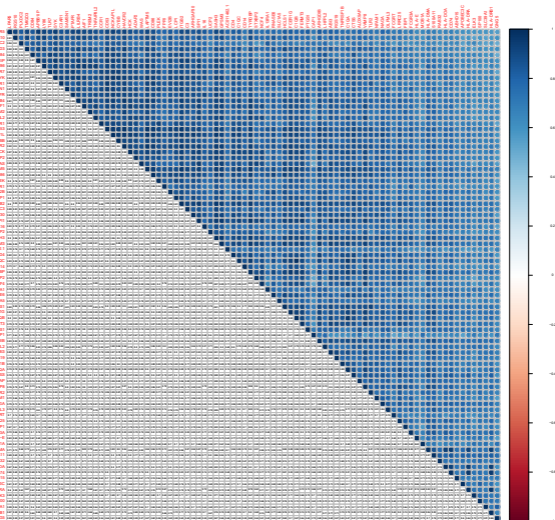

Supplement: FIGURE S4 — Analysis of key genes in the module brown. (A) The heatmap showing that the differentially expressed levels of the key genes between the normal control tissue and tumor tissue. (B) The heatmap of the correlation analysis among key genes. [file Image_4.PDF]

### Kaplan-Meier plot

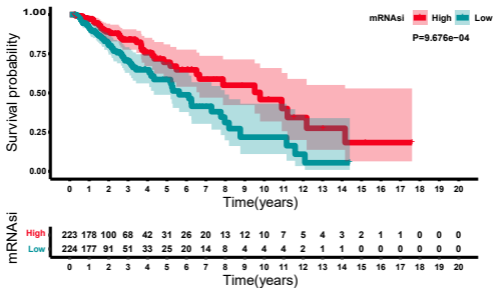

### Kaplan-Meier plot

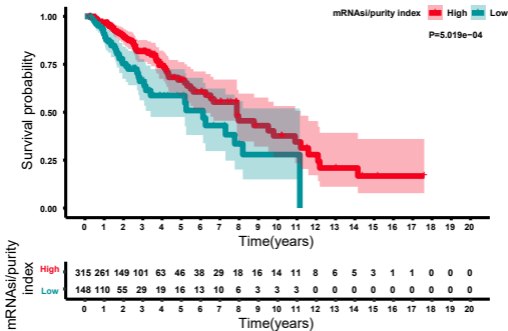

Supplement: FIGURE S5 — (A) Kaplan-Meier survival analysis of mRNAsi. (B) Kaplan-Meier survival analysis of corrected mRNAsi. Additionally, the table indicating the number at risk for each group at corresponding time points. [file Image_5.PDF]

**A**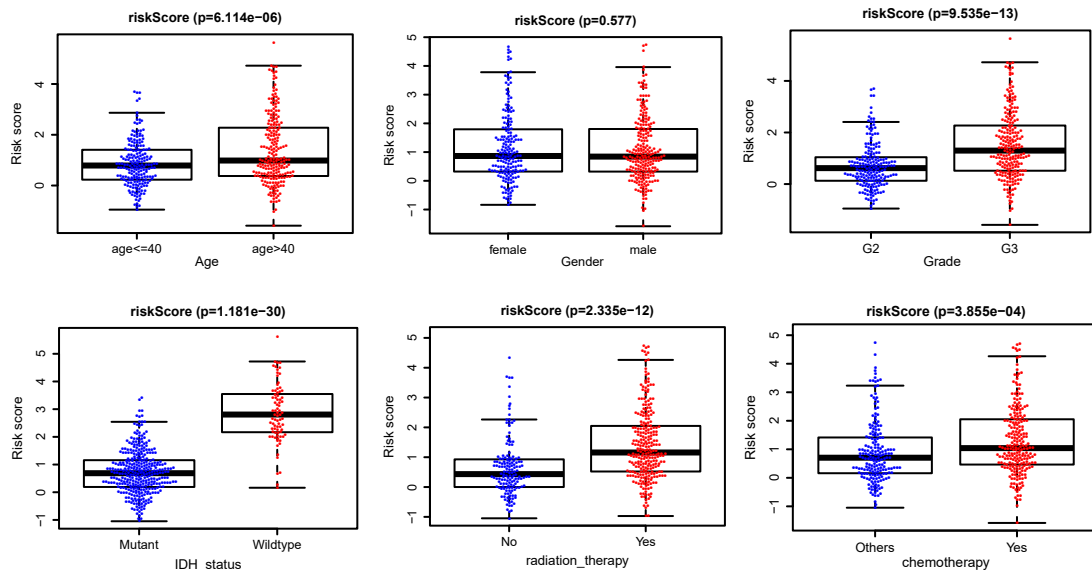**B**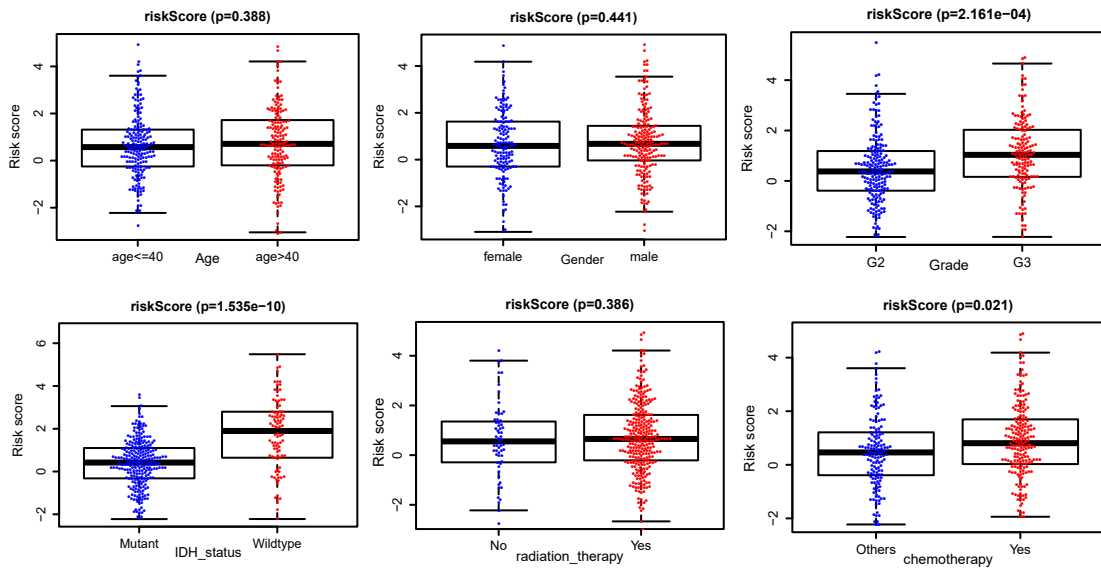

Supplement: FIGURE S6 — Association between risk score and clinical-pathological parameters. Association between risk score and age, gender, grade, radiotherapy, chemotherapy, and IDH mutation status of primary LGG patients in TCGA cohort (A), in CGGA cohort (B). [file Image_6.PDF]
