# Supplementary material for: Prognostic Value of a Stemness Index-Associated Signature in Primary Lower-Grade Glioma
Source: Front Genet. 2020 May 5;11:441. doi: 10.3389/fgene.2020.00441 (PMC7216823; doi:10.3389/fgene.2020.00441)
Supplement: FIGURE S7 — The mRNA expression level of ADAP2 (A), ALOX5AP (B), APOBEC3C (C), FCGRT (D), GNG5 (E), LRRC25 (F), and SP100 (G) in different types of human cancers. [file Image_7.PDF]

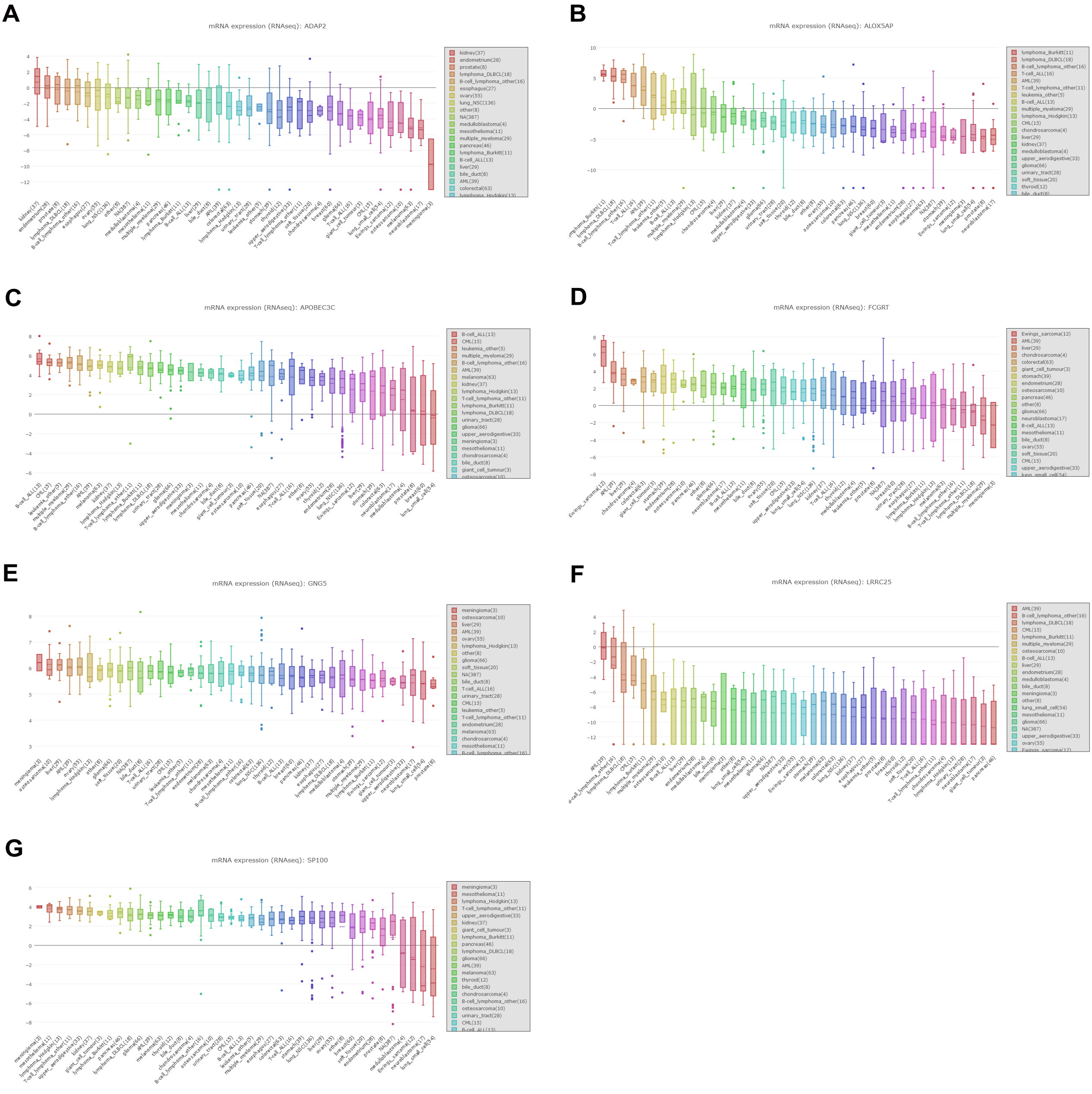
A
mRNA expression (RNAseq): ADAP2
B
mRNA expression (RNAseq): ALOX5AP
C
mRNA expression (RNAseq): APOBEC3C
D
mRNA expression (RNAseq): FCGRT
E
mRNA expression (RNAseq): GNG5
F
mRNA expression (RNAseq): LRRC25
G
mRNA expression (RNAseq): SP100
