# Supplementary material for: Prognostic Value of a Stemness Index-Associated Signature in Primary Lower-Grade Glioma
Source: Front Genet. 2020 May 5;11:441. doi: 10.3389/fgene.2020.00441 (PMC7216823; doi:10.3389/fgene.2020.00441)
Supplement: FIGURE S8 — The protein expression level of immunohistochemistry (IHC) images collected from the Human Protein Atlas database of the risk genes between glioma tissue and normal control (ADAP2 was not available). [file Image_8.PDF]

## ALOX5AP

**Normal**
Antibody HPA026592

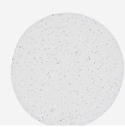

**Staining Low**

**Glioma**
Antibody HPA026592

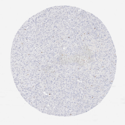

**Staining Not detected**

## APOBEC3C

**Normal**
Antibody CBA033048

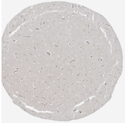

**Staining Not detected**

**Glioma**
Antibody CBA033048

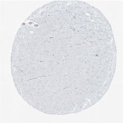

**Staining Not detected**

## FCGRT

**Normal**
Antibody HPA015130

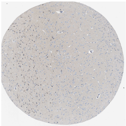

**Staining Not detected**

**Glioma**
Antibody HPA015130

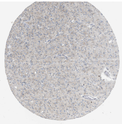

**Staining Low**

## GNG5

**Normal**
Antibody CBA032623

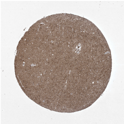

**Staining Medium**

**Glioma**
Antibody CBA032623

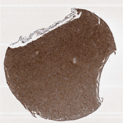

**Staining High**

## LRRC25

**Normal**
Antibody HPA029459

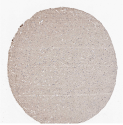

**Staining Low**

**Glioma**
Antibody HPA029459

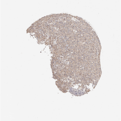

**Staining Low**

## SP100

**Normal**
Antibody HPA017384

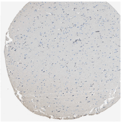

**Staining Medium**

**Glioma**
Antibody HPA017384

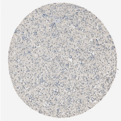

**Staining Low**
